# Assessing the Impact of COVID-19 on Antimicrobial Stewardship Activities/Programs in the United Kingdom

**DOI:** 10.3390/antibiotics10020110

**Published:** 2021-01-23

**Authors:** Diane Ashiru-Oredope, Frances Kerr, Stephen Hughes, Jonathan Urch, Marisa Lanzman, Ting Yau, Alison Cockburn, Rakhee Patel, Adel Sheikh, Cairine Gormley, Aneeka Chavda, Tejal Vaghela, Ceri Phillips, Nicholas Reid, Aaron Brady

**Affiliations:** 1 The Pharmacy Infection Network (PIN), United Kingdom Clinical Pharmacy Association (UKCPA), Leicester LE2 5BB, UK; Stephen.Hughes2@chelwest.nhs.uk (S.H.); jonathan.urch@nhs.net (J.U.); marisa.lanzman@nhs.net (M.L.); ting.yau@nhs.net (T.Y.); rakhee.patel1@nhs.net (R.P.); adel.sheikh@porthosp.nhs.uk (A.S.); aneeka.chavda1@nhs.net (A.C.); t.vaghela@nhs.net (T.V.); 2 Association of Scottish Antimicrobial Pharmacists (ASAP), Room 48, Ward 41, Regional Infectious Diseases Unit, Western General Hospital, Crewe Road, Edinburgh EH4 2XU, UK; frances.kerr@nhs.scot (F.K.); alison.cockburn@nhslothian.scot.nhs.uk (A.C.); 3 Northern Ireland Regional Antimicrobial Pharmacists Network, Medicines Optimisation Innovation Centre (MOIC), Bretten Hall, Antrim Area Hospital Site, Bush Road, Antrim BT41 2RL, UK; Cairine.Gormley@westerntrust.hscni.net (C.G.); Aaron.brady@qub.ac.uk (A.B.); 4 All Wales Antimicrobial Pharmacists Group, Cardiff CF10 4BZ, UK; ceri.phillips@wales.nhs.uk (C.P.); Nicholas.Reid@wales.nhs.uk (N.R.)

**Keywords:** COVID-19, antimicrobial stewardship (AMS), antimicrobial resistance (AMR), coronavirus, SARS-CoV-2

## Abstract

Since first identified in late 2019, the acute respiratory syndrome coronavirus (SARS-CoV2) and the resulting coronavirus disease (COVID-19) pandemic has overwhelmed healthcare systems worldwide, often diverting key resources in a bid to meet unprecedented challenges. To measure its impact on national antimicrobial stewardship (AMS) activities, a questionnaire was designed and disseminated to antimicrobialstewardship leads in the United Kingdom (UK). Most respondents reported a reduction in AMS activity with 64% (61/95) reporting that COVID-19 had a negative impact on routine AMS activities. Activities reported to have been negatively affected by the pandemic include audit, quality improvement initiatives, education, AMS meetings, and multidisciplinary working including ward rounds. However, positive outcomes were also identified, with technology being increasingly used as a tool to facilitate stewardship, e.g., virtual meetings and ward rounds and increased the acceptance of using procalcitonin tests to distinguish between viral and bacterial infections. The COVID-19 pandemic has had a significant impact on the AMS activities undertaken across the UK. The long-term impact of the reduced AMS activities on incidence of AMR are not yet known. The legacy of innovation, use of technology, and increased collaboration from the pandemic could strengthen AMS in the post-pandemic era and presents opportunities for further development of AMS.

## 1. Introduction

The novel coronavirus, SARS-CoV-2, has dominated all aspects of healthcare since it was first identified at the end of 2019 [1,2]. The coronavirus disease (COVID-19) has overwhelmed healthcare systems in those countries affected and diverted resources away from established services, as clinical teams look to manage this pandemic [3]. The antimicrobial stewardship (AMS) services, established to optimize anti-infectives and minimize the spread and impact of antimicrobial resistance (AMR), have been severely impacted by COVID-19 [4]. Whilst we battle against this pandemic, it is essential that we do not lose sight of the long-term AMR priorities.

The long-term impact of COVID-19 on AMR has been much debated in the recent literature [5,6]. The highlighted importance of infectious disease and microbiology teams in managing this emerging pandemic, the increased awareness of and use of personal protective equipment and greater focus on hand hygiene are all expected to support existing AMR strategies. Limiting patient contact and social distancing may lead to reductions in healthcare-associated transmission of disease. These benefits are likely offset by prioritized allocation of isolation rooms to COVID-19 patients over those with multi-drug resistant organisms and the reallocation of resource to fight this pandemic. Many infectious disease and microbiology teams have been repurposed to manage complex COVID-19 patients and thus established AMS services have suffered. High antibacterial prescribing in patients presenting with COVID-19 is expected to propagate AMR and presents an immediate challenge for AMR [7]. Reports of low prevalence of confirmed bacterial and fungal co-infections with COVID-19 are emerging yet high rates of empiric antibacterial prescribing are evident [8,9]. Challenges differentiating COVID-19 presentations with classical bacterial pneumonia, the established concerns with bacterial co-infection with other viral infections (e.g., influenza), and often reduced diagnostic resources all contribute to difficulties when differentiating COVID-19 from potential concurrent bacterial infection [10,11,12,13,14,15]. Understandably, in the absence of robust evidence and clear guidance, antibacterials are often added as a precaution. This is complicated further by early conflicting evidence purporting the potential antiviral role of azithromycin, subsequently leading to increased use of macrolides for non-bacterial indications [16,17,18,19].

The infection pharmacist has been central to the delivery of care on the frontline and supporting the traditional AMS role. With the increased pressure on the health system during the pandemic, infection pharmacists have been called upon as key members of the healthcare team to support and alleviate the burden on over-stretched emergency departments, intensive care units, and to support medical staff with the management of high acuity patients. In addition, AMS roles have developed in response to local needs and resource availability. Availability of new technologies and reduced patient contact have also transformed traditional services and provide unique challenges and opportunities for antimicrobial teams [20]. The expected impact of COVID-19 on existing AMS services and on antimicrobial prescribing; thus, AMR remains unknown [6].

The challenge for pharmacists to balance the demands of daily clinical duties with those of maintaining an oversight of the rapidly emerging evidence base is great. Frequent reviews of the literature, drafting local guidelines, managing the effects of fragile medication supply chains, and introducing novel anti-infective therapies within trial or compassionate use settings as well as effectively communicating these changes have become an essential role for infection pharmacists. The Pharmacy Infection Network (PIN) of the United Kingdom Clinical Pharmacy Association (UKCPAPIN) during the first wave of the pandemic in the UK sought to support pharmacists, providing peer support, and creating the opportunities for shared learning to help reduce the burden for individual pharmacists. To better understand what was being done, what the barriers were, and the potential impact of COVID-19 on existing AMS services the UKCPAPIN developed a survey for distribution to all UKCPAPIN members within the United Kingdom. The survey was purposed to explore the intended and unintended changes of AMS services, to quantify (where possible) these changes at a national level, to guide future interventions by the UKCPA to better support colleagues and advocate for relevant actions based on recommendations from the survey results.

This manuscript provides an overview of this survey, conducted in June 2020, describing the challenges and opportunities that exist in the AMS teams across the UK and Ireland and identifies how the UKCPA can better support antimicrobial pharmacists in their goals to optimize patient care in these unprecedented times.

## 2. Results and Discussion

### 2.1. Demographics of Respondents

Overall, there were responses on behalf of 95 of 169 acute trusts or health boards (56%) in the UK: 79/143 acute trusts in England, 5/14 health boards (Scotland), 7/7 health boards (Wales), and 4/5 health and social care trusts (Northern Ireland) (Table 1). This is the widest survey to date that authors can locate on the effects of the COVID-19 pandemic on AMS activities, covering almost a hundred healthcare providers (56%) across the four nations of the UK. Majority of the responding organizations were hospital trusts consisting of district/general hospitals (41%) followed by teaching hospitals (26%) (Table 1).

The approximate number of hospitalized COVID-19 cases as estimated by the respondents in the organizations (up until 31 May 2020) ranged from 0 to >2000; the majority reported having more than 500 hospitalized cases of COVID-19 at the time of the survey and four organizations reported having more than 200 hospitalized cases.

The majority of the respondents were lead antimicrobial or infection pharmacists (90%; 85/95), members of the infection/AMS pharmacy team (7%; 7/95) or microbiologist (1%; 1/95) who would have good insight into the AMS challenges and changes within their organizations. Two (2%) of the respondents were clinical pharmacists. There were no AMS nurse respondents.

### 2.2. Impact of COVID-19 on Antimicrobial Stewardship (AMS) Activities/Initiatives

When asked how much of an impact COVID-19 had had on their routine AMS activities (i.e., “In your opinion, how much impact would you say COVID-19 has had on your routine AMS activities?), 65% (61/95) felt that COVID-19 had a negative impact on routine AMS activities, with 31% (29/95) stating it had a very negative impact and 34% (32/95) describing some negative impact. While no one felt it had a very positive effect, 7% (7/95) did feel that the overall effect of COVID-19 was positive, whereas 25% (25/95) respondents thought that overall there were both positive and negative effects on AMS with the COVID-19 pandemic. Only 2 (2%) participants felt that COVID-19 had no impact on AMS activity within their hospital and one respondent stated they were unsure/unable to assess.

**Figure 1:**
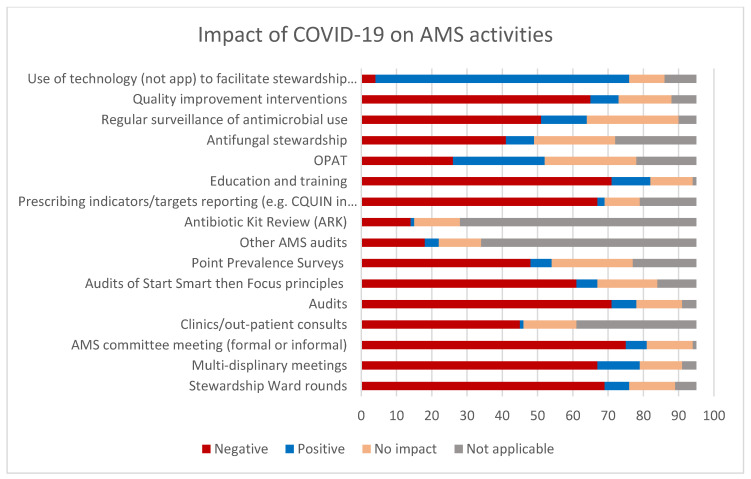
Impact of coronavirus 2019 (COVID-19) on antimicrobial stewardship (AMS) activities (*n* = 95 survey respondents).

Most of the activities listed in Figure 1 were considered to have been negatively affected by the pandemic. The greatest impact was on audit, quality improvement initiatives, education, training, AMS meetings, and multidisciplinary workings including ward rounds. Qualitative data collected through open questions also supported this, with respondents highlighting core AMS work such as reviewing and writing non-COVID-19 guidelines as being the most affected. Respondents were concerned about increased antibiotic use, including increased use of broad-spectrum antibiotics, delayed IV to oral switches (IVOST), and prolonged antibiotic durations. However, they were not able to accurately quantify increases due to the impact on routine AMS surveillance activities. In addition, there were concerns of inappropriate prescribing of antimicrobials in patients with COVID-19 infection. Although these concerns cannot be accurately quantified at present due to the UK-wide decrease in audit activities undertaken by antimicrobial pharmacists, the suspicion of increased ‘just in case’ prescribing of antimicrobials is supported by PHE Fingertips data. Analysis of this national surveillance database indicates a substantial increase in antibiotic prescribing (DDD/1000 admissions) for the current COVID-19 period in comparison to all previous quarters going back to 2017 [21]. Notably, this trend was also seen across all NHS Acute Trusts in England.

Furthermore, PHE Fingertips data also reported a reduction in the WHO-classified ‘Access’ group of antibiotics which are typically narrow-spectrum and indicated as first-line treatment agents. Conversely, an increase in both the WHO-classified ‘Watch’ and Reserve’ groups of antibiotics (typically more broad-spectrum and/or last resort antibiotics) were recorded nationally [21].

This suggests that nationwide use of antibiotics is not only increasing in overall volume but, more concerningly, in the number and volume of broad-spectrum agents prescribed. It is beyond the scope of this paper, but this trend has obvious implications for antimicrobial resistance in the months and years to

Open questions within the survey indicated that respondents were concerned that cases of *Clostridioides difficile* infection (CDI) were rising in some hospitals. It is however difficult to attribute increasing CDI rates with reduced AMS activities as there are multiple confounding factors involved. National surveillance of CDI also shows that cases were already rising pre-COVID-19 pandemic [21]. Moreover, when inquiring into what causes the increased concern for pharmacists, we found that physical limitations on conducting ward rounds, the inability to conduct regular antimicrobial audits, and the inability to see patients in person to confirm patient medication histories were most commonly cited. Stock shortages were also identified as time consuming and difficult to manage due to overwhelmed supply chains for antibiotics, antivirals and in some cases personal protective equipment (PPE). Some stock shortages for some antimicrobials such as levofloxacin appear to have commenced worldwide before the pandemic [22]. Due to the lack of routine AMS activity, it was felt that the full picture was not yet available to fully quantify the impact of COVID-19 on AMS and AMR.

Positive outcomes were also identified, with technology being increasingly used as a tool to facilitate stewardship, e.g., virtual meetings and ward rounds. The COVID-19 pandemic was also seen to break down barriers, resulting in increased collaboration. Other outcomes which respondents considered as positive were the increased introduction of novel biomarkers (e.g., procalcitonin) for differentiating viral and bacterial infections and better use of technology including virtual platforms and remote working. In addition, the use of hospital electronic prescribing systems facilitated AMS activities by antimicrobial pharmacists; allowing them to target their activities, for example identification of patients receiving excessive durations of antibiotics. There has also been a positive increase in multidisciplinary working where pharmacist contributions have been welcomed in an ever-changing evidence-based environment and pharmacists feeling valued for their contribution. Increased awareness of antimicrobial guidelines and improvements seen in infection prevention control have also been highlighted as likely to have a positive impact on AMS and resistance in the longer term. Innovation has also been key with some adapting services such as outpatient clinics and outpatient parenteral antibiotic therapy (OPAT) and changing current inpatient processes such as COVID-19 patients receiving a senior review more quickly. A virtual hospital model has been suggested as helpful to tackle the COVID-19 pandemic [23].

The majority of the respondents (73%) did not consider that there were non-COVID-19 related confounding factors that might have impacted AMS activities since the declaration of the pandemic in the UK (March 2020). For those that highlighted that there were confounding factors, these included staffing challenges within the infection team (i.e., lack of a stewardship lead microbiologist, antimicrobial pharmacists either not being in post or pharmacist AMS leads being redeployed, or needing to focus on clinical trials), drug shortages, increased post infection reviews for MRSA bacteremia and *Clostridioides difficile* cases. Positive confounding factors were also highlighted for example suspension of local meetings and national quality improvement schemes which allowed more time to review patients or target patients on high risk antibiotics.

Recently, Lynch et al. suggested that “AMS has become a casualty of the COVID-19 pandemic” [24]. In this survey we highlighted that while routine AMS activities were indeed a casualty of the COVID-19 pandemic, there were some opportunities presented and some positive outcomes. A recent review by Monnet and Harbarth reviewed the various determinants that may result in either an increase or, inversely, a decrease in AMR. They found that these determinants to be balanced [25]. However, the true impact of the COVID-19 pandemic on AMR will not become clear for months, possibly years, when full surveillance data on antimicrobial use and resistance become available. In addition, the changes in AMR will vary depending on the settings, e.g., hospital types/units (ICU vs. other units) and facilities available in these settings; the reduction in usual hospital activities (such as routine surgery), availability of electronic prescribing and stock management systems; community vs. hospital settings, the number of COVID-19 cases as well as AMS activities that continue to be implemented through the pandemic.

### 2.3. COVID-19 Specific Changes to the Management of Pneumonia

**Figure 2:**
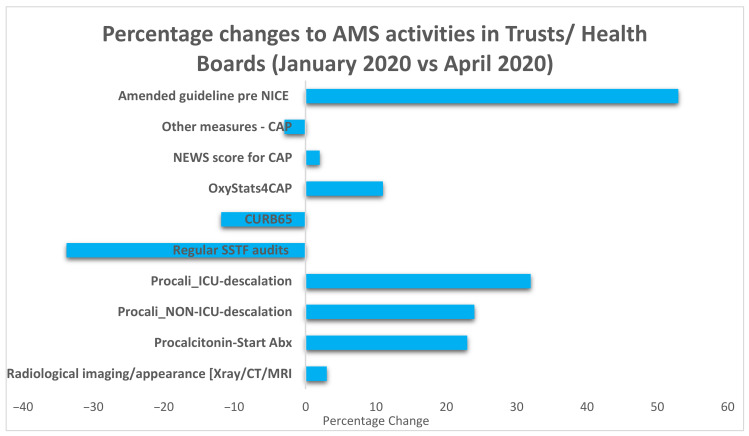
Changes to AMS initiatives as a result of the COVID-19 surge (*n* = 95). Key for the *Y*-axis: **Procalcitonin-Start Abx:** procalcitonin use to inform starting antibiotics. **Procali_NON-ICU-de-escalation:** Procalcitonin use in non-intensive care unit (ICU) settings to inform de-escalation and stopping antimicrobial stewardship activity. **Procalcitonin_ICU-de-escalation:** Procalcitonin use in ICU settings only to inform de-escalation and stopping antimicrobial stewardship activity. **Regular SSTF audits:** Regular (weekly or monthly) audit of review of antimicrobial prescriptions (Start Smart then Focus principles). **CURB65:** CURB 65 is specified in the guideline for assessing severity of Community Acquired Pneumonia **OxyStats4CAP**: Oxygen Saturations is specified in the guideline for assessing severity of Community Acquired Pneumonia. **NEWS score for CAP:** NEWS2 score is specified in the guideline for assessing severity of Community Acquired Pneumonia. **Other measures, CAP:** Other measures specified in the guideline for assessing severity of Community Acquired Pneumonia. **Radiological imaging/appearance (X-ray/CT/MRI):** Radiological imaging/appearance (X-ray/CT/MRI) to facilitate antibiotic review (de-escalating or stopping antibiotic) **Amended guideline pre NICE:** Amended antimicrobial prescribing guidance for COVID19 (pre NICE Guidance publications).

Figure 2 illustrates the identified changes in AMS activity in the management of patients with community acquired pneumonia (CAP) during the COVID-19 surge in April 2020 against a baseline of 31 January 2020. It highlights for example that the pandemic led to increased use of procalcitonin in the management of respiratory tract infection both within and outside of the ICU, guiding antibiotic de-escalation and initiation. 53% (50/95) of respondents had updated guidelines on CAP before the release of the COVID-19 rapid guideline: managing suspected or confirmed pneumonia in adults in hospital by the National Institute for Health and Care Excellence (NICE) on 1 May 2020 [26]. There was also decreased AMS monitoring through audits such as the Start Smart then Focus (SSTF) studies, and the use of the CURB65 scoring system decreased slightly. The NICE guideline for CAP highlighted that CURB65 tool for CAP had not been validated for people with COVID-19. NICE guidance for the management of pneumonia in adults in hospital specified that there is insufficient evidence to recommend routine procalcitonin testing to guide decisions about antibiotics and encouraged centers already using procalcitonin tests to participate in research and data collection [27]. However, many organizations incorporated adherence to the CURB 65 scoring and advocated use of procalcitonin within their guidelines for management of COVD-19 patients.

### 2.4. Participation in COVID-19 Clinical Trials*

At the time of the survey, almost all responding organizations (*n* = 95) were participating in the Randomised Evaluation of COVID-19 Therapy (RECOVERY) clinical trial (98%) with 75% and 58% participating in the Easy Access to Medicine Scheme (EAMS)–Remdesivir and Randomised, Embedded, Multi-factorial, Adaptive Platform Trial for Community-Acquired Pneumonia (REMAP-CAP) clinical trials respectively. Other trials and schemes taking place within responding Trusts included the since-discontinued expanded access program (EAP) for remdesivir (9%), Accelerating COVID-19 Research & Development (ACCORD-2) (8%), Safety and Efficacy of Tocilizumab in Patients With Severe COVID-19 Pneumonia (COVACTA) (6%), Platform Randomised trial of INterventions against COVID-19 In older peoPLE (PRINCIPLE) (4%), Adaptive COVID-19 Treatment Trial (ACTT) (4%), and Azithromycin versus usual care In Ambulatory COVID-19 (ATOMIC2) (2%). Two respondents reported that none of these trials were taking place in their organization. More than half of respondents also stated that their organization were part of the Early Access to Medicines Scheme (EAMS) for remdesivir.

As of 30 June 2020, there were 1142 clinical trials recruiting patients for COVID-19 management in hospitals or ICU settings globally with 62 of these registered for patients in the UK [28,29]. As perhaps expected, all organizations except two participated in the RECOVERY trial (RECOVERY; ISRCTN50189673), which was one of the two clinical trials globally that received the greatest media and scientific attention at the time. The other trial was the WHO “Solidarity” trial (ISRCTN83971151), which did not include sites in the UK. The lead role that many AMS teams had in management of these clinical trials may have contributed to the impact noted on routine AMS activities. Lack of resources for AMS because of re-allocation to COVID-19 planning and management, such as multiple trials, has also been highlighted by others [30]

### 2.5. Update of Local Guidelines and Implementation of National Guidelines

A third of responding organizations (UK-wide) had updated their local guidelines based on the NICE national guidelines for CAP and hospital-acquired pneumonia (HAP) published in April 2020; with just over 40% stating they were already aligned with the published guidelines whilst 12% of organizations stated they did not plan to update their guidelines based on national guidelines (Table 2).

**Figure 3:**
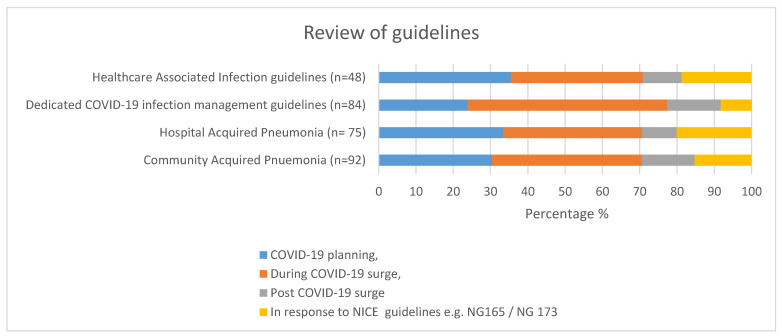
Percentage of organizations that reviewed their guidelines (*n* = 95 respondents).

A high proportion of organizations reviewed or updated their CAP, HAP, or healthcare associated infections (HCAI) guidelines as part of COVID-19 planning or during the COVID-19 surge. Three quarters of organizations (77%) also developed dedicated COVID-19 infection management guidelines (Figure 3). Other activities which had been affected during the COVID-19 first wave are highlighted in Table 3.

### 2.6. Communication Methods within Secondary Care Settings (n = 95)

Digital methods were the most common methods of communication within organizations during the COVID-19 first surge (Table 4). A variety of methods were employed to keep staff up to date with current best practice in an ever changing evidence base including the local intranet, an antibiotic app, and an increase in virtual meetings and teleconferences.

### 2.7. Staff Changes during COVID-19 Epidemic

COVID-19 has had a considerable impact on the roles and responsibilities of antimicrobial pharmacists (Table 5). More than half (57%) of antimicrobial pharmacists were seconded to other clinical roles within the pharmacy team and wider hospital with many having to undertake more than one role (Table 5). The main roles pharmacists were seconded to were ICU and general medicine. A small proportion of pharmacists also were seconded to roles outside pharmacy.

Antimicrobial pharmacists and antimicrobial pharmacy teams also undertook additional responsibilities as demonstrated in Table 6, with the highest number reporting additional responsibility for managing drug shortages, for both antimicrobial and non-antimicrobial medication. Managing supply of medication to patients with COVID-19 and providing PPE advice was also an additional role undertaken by many pharmacists during the initial COVID-19 pandemic.It is also evident that antimicrobial pharmacists had considerable involvement in the provision of infection prevention and control advice which may well have been part of the multidisciplinary ward round activities. The extension of antimicrobial pharmacists’ roles beyond traditional duties/activities has also been highlighted by Goff et al. (2020) [31]. In addition, a recent review proposed recommendation for harnessing the AMS role of pharmacists and their teams in the context of COVID-19 and importance of continuing to advocate for AMS [32].

More than half of respondents (56%) (Table 7) had to undertake additional training on their own time with only 37% being able to complete additional training and learning needs around COVID-19 within work. There was no training available for COVID-19 during the first surge as it was new to all. More than three months after the start of the pandemic, no hospital was able to provide formalized mandated training on COVID-19; this is likely to change as understanding of COVID-19 progresses and when a vaccine becomes available, which will require large scale training before administration. Learning on the job, reading papers being published from China and joining various webinars were the typical opportunities available during the surge. Our survey results showed that 92% of the respondents undertook this learning in their own time or as on the job training. This is further emphasized by the respondents in the increase in multidisciplinary team (MDT) working as the teams were learning as a team.

## 3. Materials and Methods

A quantitative survey-based approach was adopted using a 20-item questionnaire developed from the literature on AMS in the context of COVID-19 and consensus from infection/antimicrobial pharmacists (Supplementary Materials 1). Demographic data on the organization of each respondent included: which UK country, type of hospital (teaching, district/general, larger organization with multiple hospital sites, specialist), number of COVID-19 cases up until 31 May 2020, and the role of respondents. The survey was reviewed and refined by discussion with a working group comprised of members from the UKCPA Pharmacy Infection Network (UKCPAPIN), Association of Scottish Antimicrobial Pharmacists, All Wales Antimicrobial Pharmacists Group, and Northern Ireland Regional Antimicrobial Pharmacists Network. The survey was then hosted on Google Forms, a web-based survey platform, then pilot tested with five individuals across the UK. Following this initial testing, the final survey was disseminated by UKCPAPIN, Association of Scottish Antimicrobial Pharmacists, All Wales Antimicrobial Pharmacists Group, and Northern Ireland Regional Antimicrobial Pharmacists Network. The survey was also promoted via UKCPAPIN social media channels, and antimicrobial pharmacists/local network WhatsApp groups.

### 3.1. Respondent Eligibility

Pharmacy infection professionals (pharmacists, pharmacy technicians, and dispensers) across all UK secondary care and acute institutions/hospitals were the intended audience for the completion of the survey. Participation was voluntary, with the questionnaire being open for responses over a 2-week period (4 to 10 June2020).

### 3.2. Data Management

All data were held securely and in line with the General Data Protection Regulation 2016/679 (17). Study approval was also obtained from the UKCPAPIN

### 3.3. Data Analysis

Descriptive statistics on the frequency distributions and percentages were used to analyze the responses. Data were analyzed using Microsoft^®^ Excel (2010). The survey tool is provided as Supplementary Information 1.

## 4. Conclusions

The findings of our survey provides, for the first time, quantitative and qualitative data on the impact of the COVID-19 pandemic on AMS activities undertaken across the UK.

Key stewardship activities that were negatively impacted include AMS ward rounds, MDT AMS meetings, quality improvement, audits, and education/training. The long-term impact of COVID-19 and the full extent of reduced AMS activities, as well as the impact of this on AMR, is unlikely to become clear for months and possibly years. We will know more when surveillance data on antimicrobial use and resistance become available, which will likely vary depending on setting and incidence of COVID-19 within each health system. Monitoring the impact of any harm caused by reduced AMS activities such as *C. difficile*, increased multidrug resistant organisms, and increased hospital admission or length of stay and mortality further reinforced the need to preserve this vital activity in future pandemic or COVID-19 surges. An additional survey to compare the impact of AMS activity during the first wave and subsequent waves or overall would add to the evidence.

Positive impacts identified within participating organizations highlighted through the survey (linked to measures to control the pandemic) included the increased acceptance of using procalcitonin to discriminate between viral and bacterial pneumonia-reducing inappropriate antimicrobial use in viral pneumonia patients in the post pandemic era. Technology was embraced to bring some of the historic AMS activities into the digital age and should be further harnessed and promoted. Using virtual platforms for education and training, multidisciplinary team meetings, AMS meetings, AMS rounds, and virtual clinics could also continue to be encouraged for AMS activities.

While the impact of the COVID-19 pandemic on AMS activity has been quantified, the psychological impact of additional roles, secondment to other specialties, and additional responsibilities that antimicrobial pharmacists have undertaken has yet to be evaluated and may form the basis of further studies. It is important that those who lead on AMS continue to have protected time to focus on AMS during current or future pandemics.

As expected wide scale participation in clinical trials for treatments of COVID-19 have been observed across the whole of the UK. The large number of participants has contributed to and will continue to progress the understanding of treatment options for COVID-19 for the benefit of future patients.

The legacy of innovation, use of technology, and increased collaboration/links with non-infection specialists, which the pandemic made necessary, could in fact strengthen AMS in the post-pandemic era and presents further opportunities for development of the antimicrobial stewardship roles. In addition, the networking and support network that has been developed will continue to support pharmacists in this role in future.

## Figures and Tables

**Table 1:** Country distribution of responses (*n* = 95).

Country	Number of Trusts/Health Boards with Responses	% of Respondents
England	79	83.2
Scotland	5	5.3
Wales	7	7.4
Northern Ireland	4	4.2
Type of hospital/organization	Number	% of respondents
Teaching	25	26.3
District/General	39	41.1
Acute Trust with multiple types of hospitals	13	13.7
Specialist	7	7.4
Others	11	11.6
Community Trust, Mental Health Trust, or Clinical Commissioning Groups (CCG)/Primary care/Primary Care Network	0	0
Reported estimated number of COVID-19 cases by respondents	Number of respondents	% of respondents
0–50	4	4.2
51–200	10	10.5
201–500	16	16.8
501–1000	21	22.1
1000–2000	12	12.6
>2000	4	4.2
Unsure	25	26.3
Do not wish to answer	3	3.2

**Table 2:** Organizations updating guidance in line with NICE recommendations (n = 95 respondents).

National Guidelines	Yes (%)	Already Aligned (%)	Still Discussing (%)	Don’t Plan to (%)	NA (%)
Update CAP guidelines following publication of NICE NG 165 (*n* = 95)	29.5	42.1	9.5	11.6	7.4
Update HAP guidelines following publication of NICE NG173 (*n* = 95)	29.5	41.1	10.5	11.6	7.4
NICE criteria on when to stop antibiotics been implemented/promoted (*n* = 95)	36.8	27.4	24.2	5.3	6.3

**Table 3:** Other activities undertaken in organizations during the COVID-19 pandemic (*n* = 95).

Other Activities–Yes Responses	Number	%
Does your Trust have electronic prescribing for inpatients?	43	45.3
Has face to face clinical pharmacy time per patient reduced?	72	75.8
Has your organization published a specific antibiotic guideline for COVID-19?	62	65.3
Have you collected data on antibiotic use in COVID-19 patients since March 2020?	45	47.4
Is there formal recommendation/guidance/communication to stop antibiotics if patient is COVID + ve and no evidence of bacterial infection?	69	72.6
Have you collected data on bacterial co-infections since March 2020?	22	23.2

**Table 4:** Communication methods by organizations during the COVID-19 Pandemic.

Method of Communication within Organizations	Number	%
Intranet	54	56.8
Antibiotic App	50	52.6
Virtual meetings/teleconference	34	35.8
No specific cascade of messages on antibiotic use	16	16.8
Emails to staff	13	13.7
Grand rounds	13	13.7
Specific guidelines	10	10.5
Online learning, e.g., internal webinars	7	7.4

**Table 5:** New responsibilities for infection management pharmacy teams.

New Responsibilities during COVID-19 Response	Number	%
Secondment to other clinical specialties at any point for more than 0.5WTE of usual AMS activities time	54	56.8
Secondment to ICU	42	44.2
Secondment to general medicine	44	46.3
Secondment to technical services	6	6.3
Secondment to other roles within pharmacy	29	30.5
Secondment to other roles outside pharmacy	5	5.3

**Table 6:** Additional activities undertaken by AMS (*n* = 95) pharmacy teams.

Additional Organization-Wide (External to Pharmacy) Roles AMS Pharmacy Teams Were Involved in as Part of the COVID-19 Response	Number	%
Communications	67	70.5
Development of treatment guidelines linked to COVID	16	16.8
Development of other guidelines	48	50.5
Managing drug shortages (excluding antimicrobials)	29	30.5
Managing antimicrobial drug shortages	77	81.1
Monitor compliance with antimicrobial treatment guidelines	54	56.8
Management of patient’s own drugs for COVID-19 patients	53	55.8
Providing infection prevention and control advice	57	60.0
Providing personal protective equipment (PPE) advice	33	34.7
Others (wider pharmacy management responsibilities)	5	5.3

**Table 7:** Opportunity for additional learning undertaken during the COVID-19 Pandemic (*n* = 95).

Source of Significant Proportion of Learning/Training on COVID-19	Number	%
I learned on my own time	53	55.8
I have learnt on the job	35	36.8
I have not been able to dedicate time to learn about COVID-19 specifically	5	5.3
I received formal training which my hospital mandated	0	0

## Data Availability

The data presented in this study are available on request from the corresponding author. The data are not publicly available due to organisation privacy.

## Supplementary Materials

The following are available online at https://www.mdpi.com/2079-6382/10/2/110/s1. Survey Form UKCPAPIN COVID-19 Survey.
