# Supplementary material for: Assessing the Impact of COVID-19 on Antimicrobial Stewardship Activities/Programs in the United Kingdom
Source: Antibiotics (Basel). 2021 Jan 23;10(2):110. doi: 10.3390/antibiotics10020110 (PMC7912640; doi:10.3390/antibiotics10020110)
Supplement: Supplementary file 1 [file antibiotics-10-00110-s001.pdf]

UKCPAPIN COVID-19 Survey

The UKCPA PIN committee invite infection/antimicrobial pharmacists to complete this short survey which is open until 10 June
The purpose of this survey is to understand:
* the impact of the COVID-19 pandemic on antimicrobial stewardship,
* what support you believe would be helpful from UKCPAPIN or other relevant bodies

The survey should take approximately 12 minutes to complete.

Ideally we are seeking one answer per trust but if you are unsure if another colleague has completed, please do complete.

Please try to answer all questions so we can understand need and how to support or advocate for support. We will aim to publish the findings to share knowledge and support advocating for our speciality. Individual Trust names or contributors will not linked to their responses within publications.

Thank you in advance for your important contribution

UKCPAPIN committee
in collaboration with
Association of Scottish Antimicrobial Pharmacists
All Wales Antimicrobial Pharmacists Group
Northern Ireland Regional Antimicrobial Pharmacists Network
* Required

Consent

1. The survey is completely voluntary and part of UKCPA Pharmacy Infection Network support provision. You have the right to refuse to answer questions or withdraw at any time. By proceeding to the next page: I consent to UKCPAPIN collecting and using the information about me that I voluntarily provide for the purposes of the survey I have read, understand and agree to the information provided above *

Mark only one oval.

( ) Yes
( ) No

Organisation Information

2. Which UK country is your organisation in? *

Mark only one oval.

- England *Skip to question 3*
- Scotland *Skip to question 5*
- Wales *Skip to question 5*
- Northern Ireland *Skip to question 5*
- Outside UK *Skip to question 7*

English Trusts

3. Which English NHS Trust

Mark only one oval.

- Airedale NHS Foundation Trust
- Alder Hey Children's NHS Foundation Trust
- Ashford and St Peter's Hospitals NHS Foundation Trust
- Avon and Wiltshire Mental Health Partnership NHS Trust
- Barking, Havering and Redbridge University Hospitals NHS Trust
- Barnet, Enfield and Haringey Mental Health NHS Trust
- Barnsley Hospital NHS Foundation Trust
- Barts Health NHS Trust
- Basildon and Thurrock University Hospitals NHS Foundation Trust
- Bedford Hospital NHS Trust
- Berkshire Healthcare NHS Foundation Trust
- Birmingham and Solihull Mental Health NHS Foundation Trust
- Birmingham Community Healthcare NHS Foundation Trust
- Birmingham Women's and Children's NHS Foundation Trust
- Black Country Healthcare NHS Foundation Trust
- Blackpool Teaching Hospitals NHS Foundation Trust
- Bolton NHS Foundation Trust
- Bradford District NHS Foundation Trust
- Bradford Teaching Hospitals NHS Foundation Trust
- Bridgewater Community Healthcare NHS Foundation Trust
- Brighton and Sussex University Hospitals NHS Trust
- Buckinghamshire Healthcare NHS Trust
- Burton Hospitals NHS Foundation Trust
- Calderdale and Huddersfield NHS Foundation Trust
- Cambridge University Hospitals NHS Foundation Trust
- Cambridgeshire and Peterborough NHS Foundation Trust
- Cambridgeshire Community Services NHS Trust
- Camden and Islington NHS Foundation Trust
- Central and North West London NHS Foundation Trust
- Central London Community Healthcare NHS Trust

- Central London Community Healthcare NHS Trust
- Chelsea and Westminster Hospital NHS Foundation Trust
- Cheshire and Wirral Partnership NHS Foundation Trust
- Chesterfield Royal Hospital NHS Foundation Trust
- City Hospitals Sunderland NHS Foundation Trust
- Cornwall Partnership NHS Foundation Trust
- Countess Of Chester Hospital NHS Foundation Trust
- County Durham and Darlington NHS Foundation Trust
- Coventry and Warwickshire Partnership NHS Trust
- Croydon Health Services NHS Trust
- Cumbria Northumberland Tyne and Wear NHS Foundation Trust
- Dartford and Gravesham NHS Trust
- Derbyshire Community Health Services NHS Foundation Trust
- Derbyshire Healthcare NHS Foundation Trust
- Devon Partnership NHS Trust
- Doncaster and Bassetlaw Teaching Hospitals NHS Foundation Trust
- Dorset County Hospital NHS Foundation Trust
- Dorset Healthcare University NHS Foundation Trust
- Dudley and Walsall Mental Health Partnership NHS Trust
- East and North Hertfordshire NHS Trust
- East Cheshire NHS Trust
- East Kent Hospitals University NHS Foundation Trust
- East Lancashire Hospitals NHS Trust
- East London NHS Foundation Trust
- East Midlands Ambulance Service NHS Trust
- East Of England Ambulance Service NHS Trust
- East Suffolk and North Essex NHS Foundation Trust
- East Sussex Healthcare NHS Trust
- Epsom and St Helier University Hospitals NHS Trust
- Essex Partnership University NHS Foundation Trust
- Frimley Health NHS Foundation Trust
- Gateshead Health NHS Foundation Trust
- George Eliot Hospital NHS Trust
- Gloucestershire Care Services NHS Trust

- Gloucestershire Care Services NHS Trust
- Gloucestershire Health and Care NHS Foundation Trust
- Gloucestershire Hospitals NHS Foundation Trust
- Great Ormond Street Hospital for Children NHS Foundation Trust
- Great Western Hospitals NHS Foundation Trust
- Greater Manchester Mental Health NHS Foundation Trust
- Guy's and St Thomas' NHS Foundation Trust
- Hampshire Hospitals NHS Foundation Trust
- Harrogate and District NHS Foundation Trust
- Hertfordshire Community NHS Trust
- Hertfordshire Partnership University NHS Foundation Trust
- Homerton University Hospital NHS Foundation Trust
- Hounslow and Richmond Community Healthcare NHS Trust
- Hull University Teaching Hospitals NHS Trust
- Humber Teaching NHS Foundation Trust
- Imperial College Healthcare NHS Trust
- Isle Of Wight NHS Trust
- James Paget University Hospitals NHS Foundation Trust
- Kent and Medway NHS and Social Care Partnership Trust
- Kent Community Health NHS Foundation Trust
- Kettering General Hospital NHS Foundation Trust
- King's College Hospital NHS Foundation Trust
- Kingston Hospital NHS Foundation Trust
- Lancashire & South Cumbria NHS Foundation Trust
- Lancashire Teaching Hospitals NHS Foundation Trust
- Leeds and York Partnership NHS Foundation Trust
- Leeds Community Healthcare NHS Trust
- Leeds Teaching Hospitals NHS Trust
- Lewisham and Greenwich NHS Trust
- Lincolnshire Community Health Services NHS Trust
- Lincolnshire Partnership NHS Foundation Trust
- Liverpool Heart and Chest NHS Foundation Trust
- Liverpool University Hospitals NHS Foundation Trust
- Liverpool Women's NHS Foundation Trust

[illegible]
- London Ambulance Service NHS Trust
- London North West University Healthcare NHS Trust
- Luton and Dunstable University Hospital NHS Foundation Trust
- Maidstone and Tunbridge Wells NHS Trust
- Manchester University NHS Foundation Trust
- Medway NHS Foundation Trust
- Mersey Care NHS Foundation Trust
- Mid Cheshire Hospitals NHS Foundation Trust
- Mid Essex Hospital Services NHS Trust
- Mid Yorkshire Hospitals NHS Trust
- Midlands Partnership NHS Foundation Trust
- Milton Keynes University Hospital NHS Foundation Trust
- Moorfields Eye Hospital NHS Foundation Trust
- Norfolk and Norwich University Hospitals NHS Foundation Trust
- Norfolk and Suffolk NHS Foundation Trust
- Norfolk Community Health and Care NHS Trust
- North Bristol NHS Trust
- North Cumbria Integrated Care NHS Foundation Trust
- North Cumbria University Hospitals NHS Trust
- North East Ambulance Service NHS Foundation Trust
- North East London NHS Foundation Trust
- North Middlesex University Hospital NHS Trust
- North Staffordshire Combined Healthcare NHS Trust
- North Tees and Hartlepool NHS Foundation Trust
- North West Ambulance Service NHS Trust
- North West Anglia NHS Foundation Trust
- North West Boroughs Healthcare NHS Foundation Trust
- Northampton General Hospital NHS Trust
- Northamptonshire Healthcare NHS Foundation Trust
- Northern Devon Healthcare NHS Trust
- Northern Lincolnshire and Goole NHS Foundation Trust
- Northumbria Healthcare NHS Foundation Trust
- Nottingham University Hospitals NHS Trust

- Nottingham University Hospitals NHS Trust
- Nottinghamshire Healthcare NHS Foundation Trust
- Oxford Health NHS Foundation Trust
- Oxford University Hospitals NHS Foundation Trust
- Oxleas NHS Foundation Trust
- Pennine Acute Hospitals NHS Trust
- Pennine Care NHS Foundation Trust
- Poole Hospital NHS Foundation Trust
- Portsmouth Hospitals NHS Trust
- Project Nightingale NHS Trust
- Queen Victoria Hospital NHS Foundation Trust
- Robert Jones and Agnes Hunt Orthopaedic and District Hospital NHS Trust
- Rotherham Doncaster and South Humber NHS Foundation Trust
- Royal Berkshire NHS Foundation Trust
- Royal Brompton and Harefield NHS Foundation Trust
- Royal Cornwall Hospitals NHS Trust
- Royal Devon and Exeter NHS Foundation Trust
- Royal Free London NHS Foundation Trust
- Royal National Orthopaedic Hospital NHS Trust
- Royal Papworth Hospital NHS Foundation Trust
- Royal Surrey NHS Foundation Trust
- Royal United Hospitals Bath NHS Foundation Trust
- Salford Royal NHS Foundation Trust
- Salisbury NHS Foundation Trust
- Sandwell and West Birmingham Hospitals NHS Trust
- Sheffield Children's NHS Foundation Trust
- Sheffield Health and Social Care NHS Foundation Trust
- Sheffield Teaching Hospitals NHS Foundation Trust
- Sherwood Forest Hospitals NHS Foundation Trust
- Shrewsbury and Telford Hospital NHS Trust
- Shropshire Community Health NHS Trust
- Solent NHS Trust
- Somerset Partnership NHS Foundation Trust
- South Central Ambulance Service NHS Foundation Trust

- South Central Ambulance Service NHS Foundation Trust
- South East Coast Ambulance Service NHS Foundation Trust
- South London and Maudsley NHS Foundation Trust
- South Tees Hospitals NHS Foundation Trust
- South Tyneside And Sunderland NHS Foundation Trust
- South Tyneside NHS Foundation Trust
- South Warwickshire NHS Foundation Trust
- South West London and St George's Mental Health NHS Trust
- South West Yorkshire Partnership NHS Foundation Trust
- South Western Ambulance Service NHS Foundation Trust
- Southend University Hospital NHS Foundation Trust
- Southern Health NHS Foundation Trust
- Southport and Ormskirk Hospital NHS Trust
- St George's University Hospitals NHS Foundation Trust
- St Helens and Knowsley Hospitals NHS Trust
- Stockport NHS Foundation Trust
- Surrey and Borders Partnership NHS Foundation Trust
- Surrey and Sussex Healthcare NHS Trust
- Sussex Community NHS Foundation Trust
- Sussex Partnership NHS Foundation Trust
- Tameside Hospital NHS Foundation Trust
- Taunton and Somerset NHS Foundation Trust
- Tavistock and Portman NHS Foundation Trust
- Tees, Esk and Wear Valleys NHS Foundation Trust
- The Christie NHS Foundation Trust
- The Clatterbridge Cancer Centre NHS Foundation Trust
- The Dudley Group NHS Foundation Trust
- The Hillingdon Hospitals NHS Foundation Trust
- The Newcastle Upon Tyne Hospitals NHS Foundation Trust
- The Princess Alexandra Hospital NHS Trust
- The Queen Elizabeth Hospital, King's Lynn. NHS Foundation Trust
- The Rotherham NHS Foundation Trust
- The Royal Bournemouth and Christchurch Hospitals NHS Foundation Trust
- The Royal Marsden NHS Foundation Trust

- The Royal Marsden NHS Foundation Trust
- The Royal Orthopaedic Hospital NHS Foundation Trust
- The Royal Wolverhampton NHS Trust
- The Walton Centre NHS Foundation Trust
- Torbay and South Devon NHS Foundation Trust
- United Lincolnshire Hospitals NHS Trust
- University College London Hospitals NHS Foundation Trust
- University Hospital of Derby and Burton NHS Foundation Trust
- University Hospital Southampton NHS Foundation Trust
- University Hospitals Birmingham NHS Foundation Trust
- University Hospitals Bristol and Weston NHS Foundation Trust
- University Hospitals Coventry and Warwickshire NHS Trust
- University Hospitals Of Leicester NHS Trust
- University Hospitals Of Morecambe Bay NHS Foundation Trust
- University Hospitals of North Midlands
- University Hospitals Plymouth NHS Trust
- Walsall Healthcare NHS Trust
- Warrington and Halton Hospitals NHS Foundation Trust
- West Hertfordshire Hospitals NHS Trust
- West London NHS Trust
- West Midlands Ambulance Service University NHS Foundation Trust
- West Suffolk NHS Foundation Trust
- Western Sussex Hospitals NHS Foundation Trust
- Weston Area Health NHS Trust
- Whittington Health NHS Trust
- Wirral Community NHS Foundation Trust
- Wirral University Teaching Hospital NHS Foundation Trust
- Worcestershire Acute Hospitals NHS Trust
- Worcestershire Health and Care NHS Trust
- Wrightington, Wigan and Leigh NHS Foundation Trust
- Wye Valley NHS Trust
- Yeovil District Hospital NHS Foundation Trust
- York Teaching Hospital NHS Foundation Trust
- Yorkshire Ambulance Service NHS Trust

- Yorkshire Ambulance Service NHS Trust
- Other

4. Name of organisation (e.g. primary care organisation or only if not listed above)

____

Skip to question 8

Scotland, Wales and Northern Ireland health boards or trusts

5. Which Health Board or Trust

Mark only one oval.

- NHS Ayrshire & Arran
- NHS Borders
- NHS Dumfries & Galloway
- NHS Fife
- NHS Forth Valley
- NHS Grampian
- NHS Greater Glasgow and Clyde
- NHS Highland
- NHS Lanarkshire
- NHS Lothian
- NHS Orkney
- NHS Shetland
- NHS Tayside
- Velindre University NHS Trust
- NHS Eileanan Siar Western Isles
- Aneurin Bevan University Health Board
- Betsi Cadwaladr University Health Board
- Cardiff and Vale University Health Board
- Cwm Taf Morgannwg University Health Board
- Hywel Dda University Health Board
- Powys Teaching Health Board
- Swansea Bay University Health Board
- Belfast HSC Trust,
- South Eastern HSC Trust,
- Western HSC Trust,
- Southern HSC Trust
- Northern HSC Trust

6. Name of organisation (e.g. primary care organisation or only if not listed above)

____

Skip to question 8

Country

7. What country is your hospital based in?

____

More about your role and Trust

8. Indicate what type of hospitals/organisation this is? If primary care, please select other and specify name of organisation *

Mark only one oval.

- ( ) District/General
- ( ) Teaching
- ( ) Specialist
- ( ) Acute Trust with multiple types of hospitals
- ( ) Community Trust
- ( ) Mental Health Trust
- ( ) CCG/Primary care/Primary Care Network
- ( ) Other: _______________________________

9. What is your role? *

Mark only one oval.

- Lead Antimicrobial/Infection Pharmacist
- Member of Infection/Antimicrobial stewardship pharmacy team
- AMS nurse
- Other: ______________________________

10. What is the approximate number of hospitalised COVID cases in your Trust/health board (up until 31 May 2020)

Mark only one oval.

- 1-50
- 51-200
- 201 – 500
- 501 – 1000
- 1000 - 2000
- > 2000
- Unsure
- Do not wish to answer

AMS activities

11. 5. Please indicate which of the following AMS initiatives/activities were in place a) 31 January 2020 b) 30 April 2020. Please provide a response for each one that is applicable. BLA response for Jan or April 2020 will be taken as not in place. Please only select NOT applicable if e.g in primary care for PCT. *

Check all that apply.

	January 2020	April 2020	Not in place at either time	Not applicable
Radiological imaging/appearance [Xray/CT/MRI] to facilitate antibiotic review (de-escalating or stopping antibiotic)	☐	☐	☐	☐
procalcitonin use to inform STARTING antibiotics	☐	☐	☐	☐
Procalcitonin use in NON ICU settings to inform de-escalation and stopping abx stewardship activity	☐	☐	☐	☐
Procalcitonin use in ICU settings ONLY to inform de-escalation and stopping abx stewardship activity	☐	☐	☐	☐
Regular (weekly or monthly) audit of review of antimicrobial prescriptions (Start Smart then Focus principles)	☐	☐	☐	☐
CURB 65 is specified in the guideline for assessing severity of Community Acquired Pneumonia	☐	☐	☐	☐
Oxygen Sats is specified in the guideline for assessing severity of Community Acquired Pneumonia	☐	☐	☐	☐
NEWS2 score is specified in the guideline for assessing severity of Community Acquired Pneumonia	☐	☐	☐	☐
Other measures specified in the	☐	☐	☐	☐

guideline for assessing severity of Community Acquired Pneumonia				
Amended antimicrobial prescribing guidance for COVID-19 (pre NICE Guidance publications)	☐	☐	☐	☐

12. You can use this section to provide context

____
____
____
____
____

13. In your opinion, how much impact would you say COVID-19 has had on your routine AMS activities *

Mark only one oval.

- No impact
- Very negative impact
- Some negative impact
- Neither negative or positive
- Positive impact
- Very positive impact
- A mix of positive and negative impact
- Unsure/unable to assess

14. What has been the most helpful/positive impact of COVID-19 on AMS activity please explain in what way it helpful/positive

15. What has been the most detrimental/negative impact of COVID-19 on AMS activity/What, if anything, hasn’t worked so well? Please explain in what way it has been detrimental/negative

16. Which AMS activities have been impacted *

Mark only one oval per row.

	Positive	Negative	No impact	Not applicable
Stewardship Ward rounds				
Multi-displinary meetings				
AMS committee meeting (formal or informal)				
Clinics/out-patient consults				
Audits				
• Audits of Start Smart then Focus principles				
• Point Prevalence Surveys				
• Others				
Antibiotic Kit Review (ARK)				
Prescribing indicators/targets reporting (e.g. CQUIN in England)				
Education and training				
OPAT				
Antifungal stewardship				
Regular surveillance of antimicrobial use				
Quality improvement interventions				
Use of technology (not app) to facilitate stewardship (e.g. Zoom, MS Teams, Skype)				
Others (pls specify below)				

17. Please use this comment box to specify other AMS activities impacted including if positive, negative or no impact

18. Have there been any non-COVD-19 related confounding factors that might have impacted AMS activities since March?

Mark only one oval.

- Yes
- No

19. Please use this section to share new practices/beneficial innovations related to AMS which have commenced as result of COVID-19 challenges. Providing details of the current or intended impact would also be very helpful

20. Please provide further details if yes to non-COVID-19 comfounding factors impacting AMS

21. Has your organisation participated in any of the following clinical trials/experimental/expanded access programme for treatment of adult COVID patients? (tick all that apply)

Check all that apply.

- [ ] RECOVERY
- [ ] PRINCIPLE
- [ ] REMAP-CAP
- [ ] ATOMIC2
- [ ] COVACTA
- [ ] ACTT
- [ ] ACCORD-2
- [ ] Expanded Access Programme (EAP)– Remdesivir (discontinued)
- [ ] Easy Access to Medicine Scheme (EAMS) - Remdesivir
- [ ] Non applicable
- [ ] Other, please specify

22. Implementation of national guidelines *

Mark only one oval per row.

	Yes	No – we were already partially or fully aligned	No – we do not plan to	Not yet, we are currently discussing	Not applicable (e.g. Scotland, Wales & NI)
Have you updated your community acquired pneumonia guidelines following publication NG 165 ?	⬭	⬭	⬭	⬭	⬭
Have you updated your hospital pneumonia guidelines following publication of NICE NG173?	⬭	⬭	⬭	⬭	⬭
Has the NICE criteria on when to stop antibiotics been implemented/promoted?	⬭	⬭	⬭	⬭	⬭
Have you updated guidelines based on SIGN or SAPG in Scotland or AWMSG, AWAGG or RHIG in Wales?	⬭	⬭	⬭	⬭	⬭

23. Which parts of usual antimicrobial guidelines have been reviewed during COVID-19

Check all that apply.

	Pre COVID-19 planning,	During COVID-19 surge,	Post COVID-19 surge	In response to NICE guidelines e.g. NG165 / NG 173
Community Acquired Pnuemonia	☐	☐	☐	☐
Hospital Acquired Pneumonia	☐	☐	☐	☐
Dedicated COVID-19 infection management guidelines	☐	☐	☐	☐
Healthcare Associated Infection guidelines	☐	☐	☐	☐

24. Other activities *

Mark only one oval per row.

	Yes	No	Unsure
Has your organisation published a specific antibiotic guideline for Covid?			
Has face to face clinical pharmacy time per patient reduced			
Have you collected data on bacterial co-infections since March 2020			
Have you collected data on antibiotic use in COVID-19 patients since March 2020			
Does your Trust have electronic prescribing for inpatients?*			
Is there formal recommendation /guidance/communication to stop antibiotics if patient is COVID +ve and no evidence of bacterial infection?			
During the COVID response, have some or all of antimicrobial pharmacy team members been seconded to other clinical specialities at any point for more than 0.5WTE of their usual AMS activities time			
Secondment to ICU			
Secondment to general medicine			
Secondment to technical services			
Secondment to other roles within pharmacy			
Secondment to other roles outside pharmacy			

25. Which of the following activities has the antimicrobial stewardship/infection pharmacist(s) been involved in as part of the Trust wide response/preparation (i.e external to pharmacy)? (tick all that apply)

Check all that apply.

- [ ] Communications
- [ ] Development of treatment guidelines linked to COVID
- [ ] Development of other guidelines
- [ ] Managing drug shortages (excluding antimicrobials)
- [ ] Managing antimicrobial drug shortages
- [ ] Monitor compliance with antimicrobial treatment guidelines
- [ ] Management of PODs for COVID patients
- [ ] Providing infection prevention and control advice
- [ ] Providing PPE advice
- [ ] None

Other: [ ] ____________________

26. What is your single greatest stewardship concern right now/for the near future as a result of COVID-19 impact? If none, please state this

____

____

____

____

____

Education and Training

27. Which of these reflect the significant proportion of your training on COVID-19?

Mark only one oval.

- ( ) I have not been able to dedicate time to learn about COVID-19 specifically
- ( ) I learned on my own time;
- ( ) I have learnt on the job
- ( ) I received formal training which my hospital mandated

28. What training resources have you used or accessed on COVID-19. (Tick all that apply)

Check all that apply.

- [ ] NES Scotland/HEE
- [ ] RPS
- [ ] UKCPAPIN/RPS – infection Q & A webinars
- [ ] UKCPAPIN discussion forum
- [ ] "Antimicrobial Pharmacists" Whatsapp group
- [ ] Other discussion forums
- [ ] PHE/AWTTC/SAPG
- [ ] Specialist Pharmacists Services (SPS)
- [ ] Others

29. How have key messages about antibiotic use been communicated internally within your Trust/health board since 01 March 2020

Check all that apply.

- [ ] No specific cascade of messages on antibiotic use
- [ ] Intranet
- [ ] Emails to staff
- [ ] Specific guidelines
- [ ] Grand rounds
- [ ] Online learning e.g internal webinars
- [ ] Virtual meetings/teleconference
- [ ] Antibiotic App
- [ ] Others, specify

Other: [ ] ____________________

Other infection related questions

The following are not direct AMS questions, however we know that infection pharmacists have been asked to lead on or advice on the following

30. How were/are PODs of COVID-19 positive patients handled.

Check all that apply.

	During COVID-19 surge - Mid April 2020	01 June 2020	Not implemented at either time point
Used on the wards (where suitable/applicable)	☐	☐	☐
Discarded at ward level	☐	☐	☐
Returned to pharmacy for discarding	☐	☐	☐
Quarantined for a period before use	☐	☐	☐
Packages disinfected and used where suitable	☐	☐	☐
Other	☐	☐	☐

31. Are you aware of the new handling medicines guidance, IPC recommendations published on SPS website and has your trust started considering implementation?

Mark only one oval.

- Yes aware and already started implementing
- Yes aware but we were already aligned
- Yes aware but not yet started implementing
- Not aware of it

32. If appropriate, please provide further details on PODs management in the context of COVID

____

____

____

____

____

33. Training of pharmacy team on relevant PPE for their area of work

Mark only one oval.

- All patient facing pharmacy staff received training routinely as of January 2020
- All relevant patient facing pharmacy staff have now received training on PPE since March 2020
- Other: _______________

Support from UKCPAPIN

34. How did the UKCPAPIN support your practice during COVID-19 surge

____

____

____

____

____

35. How can UKCPAPIN continue to support your practice moving forward

36. Would you be interested in a joint project (e.g. PPS on COVID patients) with other UKCPAPIN members?

Mark only one oval.

- Yes
- No
- Possibly
- Other: ____________

37. If interested in contributing to joint project, please provide your email address here

38. Do you regularly (at least monthly) view the UKCPAPIN forum on UKCPA website?

Mark only one oval.

- Yes
- No

39. Are there any key learning points you wish to share? Please provide details in this section and if you wish, include your name and role so we can acknowledge you as part of shared learning

40. Can we keep in touch with you? E.g. to share webinars, forth coming masterclasses. If yes, please type your email address in this section. If you provided in earlier question, state as above or provide an alternative email address.

41. Any other comments and/or questions?

This content is neither created nor endorsed by Google.

Google Forms
